# The “care” protocol: the role of personality in a three-year follow-up study of medication overuse headache

**DOI:** 10.1186/1129-2377-14-S1-P174

**Published:** 2013-02-21

**Authors:** F Galli, G Sances, N Ghiotto, A Frustaci, E Guaschino, G Nappi, C Tassorelli

**Affiliations:** 1 Headache Science Centre of the IRCCS ‘National Institute of Neurology C. Mondino’ Foundation, Pavia, Italy, Italy; 2 Clinical and Molecular Epidemiology, IRCCS San Raffaele Pisana, Rome, Italy, Italy

## Introduction

The negative prognostic value of psychiatric disorders in Medication Overuse Headache [1] has been previously outlined, however, to the best of our knowledge, the role of personality factors as potential predictors of MOH evolution has never been studied. Aim of this study was to analyse the role of personality in the prognosis of MOH.

## Methods

Among a total of 243 patients, 150 completed the follow-up at three years (79.3% females, age 46.40±11.31). The personality profile was assessed with the Minnesota Multiphasic Personality Inventory (MMPI-2). We explored the occurrence (or not) of at least one episode of drug overuse taking into account the overall 3-year period of follow-up. Our population was subdivided into 3 groups: Group A (patients who never stopped overusing drugs after the initial detoxification treatment (N=13)); Group B (patients who stopped drug overuse following detoxification, but then relapsed at least once (N=38)); Group C (stopped drug overuse following detoxification and never relapsed (N=99)).

## Results

As regards personality profile at MMPI-2, subjects in Group A had higher scores at the Lie scale (p=0.004) as compared to both the other groups (B and C), and at the following scales as compared to patients who stopped abuse and never relapsed (Group C): Frequency (p=0.020), Hypocondriasis (p=0.007), Depression (p=0.003), Paranoia (p=0.025), Fears (p=0.003), Obsessiveness (p=0.026), Bizarre Mentation (p=0.046), Social Discomfort (p=0.004), Negative Treatment Indicators (p=0.040), Repression (p=0.007), Overcontrolled Hostility (p=0.040), Addiction Admission Scale (p=0.021), Social Responsibility(p=0.039) and Marital Distress (p=0.028).

## Conclusions

Personality is important not only because they characterise patients with MOH, but also probably for their outcome predicting value. We provide support for the existence of a small sub-group of MOH patients (Group A) with addiction-related personality and behavioural problems that are likely to play a major role in influencing and nurturing drug abuse and chronic headache.

## References

[B1] Hagen K Linde M Steiner TJ Stovner LJ Zwart JA Risk factors for medication-overuse headache: An 11-year follow-up study. The Nord-Trondelag Health Studies Pain 2011 14 1 56 61 22018971 10.1016/j.pain.2011.08.018

